# Hepatitis C Virus Core Protein Induces Neuroimmune Activation and Potentiates Human Immunodeficiency Virus-1 Neurotoxicity

**DOI:** 10.1371/journal.pone.0012856

**Published:** 2010-09-21

**Authors:** Pornpun Vivithanaporn, Ferdinand Maingat, Liang-Tzung Lin, Hong Na, Christopher D. Richardson, Babita Agrawal, Éric A. Cohen, Jack H. Jhamandas, Christopher Power

**Affiliations:** 1 Division of Neurology, University of Alberta, Edmonton, Alberta, Canada; 2 Department of Pharmacology, Faculty of Science, Mahidol University, Bangkok, Thailand; 3 Department of Microbiology and Immunology, Dalhousie University, Halifax, Nova Scotia, Canada; 4 Department of Surgery, University of Alberta, Edmonton, Alberta, Canada; 5 Institut de recherches cliniques de Montréal (IRCM) and Department of Microbiology and Immunology, University of Montreal, Montreal, Quebec, Canada; University of Toronto, Canada

## Abstract

**Background:**

Hepatitis C virus (HCV) genomes and proteins are present in human brain tissues although the impact of HIV/HCV co-infection on neuropathogenesis remains unclear. Herein, we investigate HCV infectivity and effects on neuronal survival and neuroinflammation in conjunction with HIV infection.

**Methodology:**

Human microglia, astrocyte and neuron cultures were infected with cell culture-derived HCV or exposed to HCV core protein with or without HIV-1 infection or HIV-1 Viral Protein R (Vpr) exposure. Host immune gene expression and cell viability were measured. Patch-clamp studies of human neurons were performed in the presence or absence of HCV core protein. Neurobehavioral performance and neuropathology were examined in HIV-1 Vpr-transgenic mice in which stereotaxic intrastriatal implants of HCV core protein were performed.

**Principal Findings:**

HCV-encoded RNA as well as HCV core and non-structural 3 (NS3) proteins were detectable in human microglia and astrocytes infected with HCV. HCV core protein exposure induced expression of pro-inflammatory cytokines including interleukin-1β, interleukin-6 and tumor necrosis factor-α in microglia (*p*<0.05) but not in astrocytes while increased chemokine (e.g. CXCL10 and interleukin-8) expression was observed in both microglia and astrocytes (*p*<0.05). HCV core protein modulated neuronal membrane currents and reduced both β-III-tubulin and lipidated LC3-II expression (*p*<0.05). Neurons exposed to supernatants from HCV core-activated microglia exhibited reduced β-III-tubulin expression (*p*<0.05). HCV core protein neurotoxicity and interleukin-6 induction were potentiated by HIV-1 Vpr protein (*p*<0.05). HIV-1 Vpr transgenic mice implanted with HCV core protein showed gliosis, reduced neuronal counts together with diminished LC3 immunoreactivity. HCV core-implanted animals displayed neurobehavioral deficits at days 7 and 14 post-implantation (*p*<0.05).

**Conclusions:**

HCV core protein exposure caused neuronal injury through suppression of neuronal autophagy in addition to neuroimmune activation. The additive neurotoxic effects of HCV- and HIV-encoded proteins highlight extrahepatic mechanisms by which HCV infection worsens the disease course of HIV infection.

## Introduction

Hepatitis C virus (HCV) infects approximately 180 million people worldwide [1] while 30% of individuals infected with human immunodeficiency virus type 1 (HIV-1) are co-infected with HCV due to similar routes of transmission [2]. Epidemiological studies suggest that HCV co-infection is associated with accelerated HIV disease progression, worsened clinical outcomes and increased mortality [2], [3]. HIV/HCV co-infected patients have higher HCV levels and lower likelihood of spontaneous HCV clearance, together with faster progression to liver cirrhosis [1], [2]. HCV is a member of the *Flaviviridae* family, which consists of several neurotropic viruses including St Louis encephalitis virus, Dengue and West Nile virus [4], [5]. HCV mono-infected and HIV/HCV co-infected individuals display neuropsychological deficits indicative of impaired cognition [5], [6], [7]. Magnetic resonance spectroscopy studies report alterations in cerebral metabolites among HCV-infected individuals correlated with neurocognitive impairment, including suppression of the neuronal marker, *N*-acetyl aspartate [8].

HCV transcripts and proteins have also been detected in brains from HIV/HCV co-infected patients, indicating that HCV is neuroinvasive [9], [10], [11]. The negative strand of HCV RNA, a viral replication intermediate, has been detected in the brain and cerebrospinal fluid [10], [12]. Several studies showed that HCV RNA sequences isolated from the central nervous system (CNS) were closely related to those found in peripheral blood mononuclear cells (PBMC) but were phylogenetically different from serum- or liver-derived sequences [10], [11], [12], [13], leading to the postulation that HCV enters the brain through the ‘Trojan horse’ mechanism similar to HIV-1 [4]. However, the mechanisms by which HCV exerts neuropathogenic effects remain unknown and the understanding of combined neuropathogenesis of HCV and HIV-1 is limited.

HIV-1 exerts its direct neurotoxic effects through several secreted proteins including gp120, Tat, Nef and Vpr [14]. HIV-1 Viral Protein R (Vpr) triggers neuronal apoptosis and transgenic mice expressing Vpr in brain monocytoid cells display neuronal injury as well as neurobehavioral deficits [15]. Resident brain macrophages or microglia infected with HIV-1 or exposed to HIV-encoded proteins secrete proinflammatory cytokines e.g. interleukin-1β (IL-1β) and tumor necrosis factor-α (TNF-α) and chemokines e.g. CXCL10 and CXCL12, which cause neuronal death and pathogenic immune responses in the brain [14]. Suppression of neuronal autophagy by retroviral infections has been highlighted as a putative mechanism leading to neuronal cell death and neurodegeneration. The increased level of p62 transcript and the reduction of light chain 3 type II (LC3-II), markers of autophagy inhibition, were detected in brain tissues of patients with HIV-associated dementia [16], [17].

Recently, HCV proteins including core, non-structural protein 3 (NS3) and NS5A were detected in macrophages/microglia and astrocytes but not in neurons nor oligodendrocytes of patients with HIV/HCV co-infection [9], [18]. Nevertheless, the underlying mechanisms by which HCV infects macrophages/microglia or astrocytes remain unclear. The HCV JFH1 clone, derived from a Japanese individual with fulminant hepatitis, replicates and produces infectious virus in Huh 7.5 hepatocytes [19], [20]. The entry of HCV into cells involves several membrane receptors including the scavenger receptor class B type I (SR-BI), the tetraspanin CD81, and the tight-junction molecules, claudin-1 and occludin [21]. Given these receptors are expressed by microglia and astrocytes in human and mouse brains [22], [23], [24], [25], [26], these cells are potentially permissive to infection by HCV.

The most abundantly expressed HCV protein, core, is released and soluble in blood as a part of HCV morphogenesis [27] and serum levels of HCV core protein ranges from pg/ml to ng/ml [28]. HCV core protein is cytotoxic to hepatocytes through death-receptor mediated apoptosis [29], [30]; conversely, other studies have demonstrated that HCV core protein has anti-apoptotic effects in hepatocytes [29]. Recombinant HCV core protein fused to β-galactosidase induces the production of inflammatory cytokines, e.g. interleukin-6 (IL-6) and tumor necrosis factor-α (TNF-α) in monocytes [31] as well as the chemokine CXCL8 in monocytes [31] and lung fibroblasts [32]. Additionally, HCV core protein has been implicated in suppressing differentiation, proliferation and function of T cells, dendritic cells and macrophages [33]. Herein, we investigated cell culture-derived HCV (HCV^cc^) infection of astrocytes and microglia as well as the effects of HCV core protein on neurons, astrocytes and microglia in the presence or absence of HIV-1 Vpr protein. The present results demonstrated that HCV core protein caused immune activation of glial cells and was neurotoxic in an additive manner with the HIV-1 Vpr protein.

## Materials and Methods

### Reagents

Recombinant HCV core protein fused to β-galactosidase (Gal-core, genotype 1b) and β-galactosidase (Gal) were obtained from Virogen (Watertown, MA, USA). The full-length recombinant HIV-1 Vpr protein was prepared as previously described [15].

### Standard Protocol Approvals, informed consents and ethic statements

The use of autopsied brain tissues and blood were approved under the protocol number 2291 by the University of Alberta Human Research Ethics Board (Biomedical) and written informed consents were signed before or at the collection time. Human fetal tissues were obtained from 15–19 week aborted fetuses with written consent approved under the protocol 1420 by the University of Alberta Human Research Ethics Board (Biomedical). All animals were housed and monitored on a regular schedule immediately following surgical implantation and behavioral tests according to the Center For Animal Care and Control Guidelines. This study was approved under the protocol number 452 by the University of Alberta Animal Care & Use Committee for Health Sciences.

### Cell culture

Human fetal neurons were prepared and cultured in the presence of cytosine arabinoside [34] while human fetal astrocytes (HFAs) and human fetal microglia (HFµφ) were grown in the absence of cytosine arabinoside. Microglia were collected from supernatants at 7 to 10 days after isolation while astrocytes were split and used from the fifth to the tenth passage [35].

### Human tissue samples

Human white matter, cortex, basal ganglion and spleen were collected at autopsy and stored at −80°C. Non HIV-infected controls were comprised of other neurological diseases including Alzheimer's disease, multiple sclerosis and stroke [16], [36].

### Hepatitis C viral preparation and infection

J6/JFH plasmid is a gift from Dr. Charles Rice (The Rockefeller University, New York) [19]. The HCV-J6/JFH infectious virus was prepared in Huh7.5 cells, as previously described [37]. HFAs and HFµφ were infected with supernatant containing HCV^cc^ overnight before replacing with fresh media.

### HIV-1 cultures

Human peripheral blood mononuclear cells (PBMCs) were purified from healthy subjects' blood with Histopaque (Sigma, Oakville, Ontario, CA) [38]. Peripheral blood lymphocytes (PBLs) were isolated from PBMCs and maintained in RPMI 1640 medium with 15% FBS with PHA-P stimulation for 3 days, followed by hIL-2 stimulation. At day 3 post-isolation, PBLs were infected with a neurotropic HIV-1 strain (HIV-1 SF162) and supernatants were collected at day 7 and 10 post-infection.

### Immunofluorescence

HFNs, HFAs and HFµφ were plated on poly-L-ornithine (Sigma) coated coverslips or Lab-Tex chambered coverglass (Nunc, Rochester, NY). After 72 hr exposure to HCV^cc^, HFµφ and HFAs were fixed, permeabilized and immuno-labelled with mouse anti-HCV core (clone 11-B3, GeneTex, Irvine, CA) or mouse anti-HCV NS3 (ab65407, Abcam, Cambridge, MA) and rabbit anti-ionized calcium binding adaptor molecule 1 (Iba-1, Wako Chemicals, Neuss, Germany) in HFµφ or rabbit anti-glial fibrillary acidic protein (GFAP, DAKO, Denmark) for HFAs. After 48 hr of exposure to either Gal or Gal-core proteins, HFNs were fixed, permeabilized and stained with antibodies to microtubule-associated protein-2 (MAP-2, Sigma) and LC3 (Novus Biological, Littleton, CO). Paraffin-embedded sections (6 µm) of mice brain tissue were deparaffinized and hydrated followed by antigen retrieval with boiled 0.01 M citrate buffer, pH 6.0, for 10 min. Brain sections were immunostained with anti-GFAP, anti-Iba-1 or anti-LC3 antibodies. After primary antibody incubation, cells and tissue sections were exposed to species-specific secondary antibodies labeled with Alexa Flour 488 (Invitrogen, Eugene, OR) and/or Cy3 (Jackson ImmunoResearch, West Grove, PA) [15]. Images were collected using an inverted LSM510 Meta confocal microscope.

### Real-time RT-PCR

HFµφ and HFAs in 6-well plates were exposed to either Gal or Gal-core proteins for 12 hr or as stated. RNA was extracted with TRIzol (Invitrogen) and purified by RNeasy mini columns (Qiagen, Mississauga, Ontario, Canada). First-strand complementary DNA (cDNA) was synthesized from 1 µg of total RNA mixed with random hexamer primers (Roche) and Superscript II reverse transcriptase (Invitrogen) according to the manufacturer's recommended protocols. Semiquantitative real-time PCR was performed using Bio-Rad iQ SYBR green supermix (Bio-Rad, Hercules, CA) on Bio-Rad iQ5. All PCR primers are shown in Table S1. Data were normalized to GAPDH mRNA levels and expressed as relative fold increases compared with controls ± SEM. For the positive-strand or negative-strand assays, antisense HCV-114R (5′-GAGGCTGCACGACACTCATACT-3′) and sense primer HCV-20F (5′-CGACACTCCACCATGAATCACT-3′) were used for cDNA synthesis [39]. Platinum® Quantitative PCR SuperMix-UDG and TaqMan probe HCV-P43 (5′FAM-CCCTGTGAGGAACTACTGTCTTCAC-GCAGA-TAMRA3′) were used for strand-specific real-time PCR.

### Enzyme-linked immunosorbent assay (ELISA)

Supernatants from Gal- or Gal-core treated HFAs were collected at 6, 12, 24 and 48 hr after treatments and amount of CXCL8 was measured using a human CXCL8 ELISA Ready-SET-Go (eBioscience, San Diego, CA).

### Electrophysiological studies

Whole-cell patch-clamp recordings from HFNs were performed under voltage-clamp conditions [15].

### Western blotting

6 hr after Gal or Gal-core exposure, HFNs were lysed in lysis buffer [20 mM Tris, 1% NP-40, 50 mM NaCl, Protease Inhibitor cocktail set III (1∶1000, Calbiochem, La Jolla, CA)]. Crude protein lysates were separated by 16% SDS-PAGE and the membrane were blotted for LC3 and β-actin (Santa Cruz Biotechnology, Santa Cruz, CA). Immunoreactive bands were visualized using HRP-conjugated secondary antibodies and quantitated using Quantity One™ (Bio-Rad) imaging software.

### *In vitro* cytotoxicity assay

HFNs or HFAs were plated at 5×10^4^ and 2×10^4^ cells in 96-well flat bottom plates and exposed to either Gal or Gal-core protein with and without Vpr. After 48 h of exposure, cells were fixed, permeabilized and stained with anti-β-tubulin antibodies (Sigma). Neuronal injury was quantified by β-III-tubulin immunoreactivity using Odyssey® Imager (LI-COR, Lincoln, NE). Diminished β-III-tubulin immunoreactivity in neurons is an indicative of reduced cellular viability including neurite retraction and impaired survival [15].

### Neurobehavioral studies

Vpr transgenic mice express Vpr under the control of the *c-fms* (M-CSF receptor) promoter, driving transgene expression chiefly in monocytoid cells [40]. *In vivo* neurological injury was assessed using the Ungerstedt model [41], [42] in Vpr transgenic mice. Female animals (4 weeks, n = 6) were placed in a stereotaxic frame under Ketamine/Xylazine anesthesia. The coordinates of implantation were 3.5 mm posterior, 2.5 mm lateral and 3 mm deep relative to the bregma. 2 µl of Gal or Gal-core was stereotactically implanted into the right striatum of mice. Ipsiversive rotation number was counted over 10 min after intraperitoneal injection of amphetamine (1 mg/kg) on days 4, 7 and 14 following intrastriatal injection.

### Neuronal count

After deparaffinization and hydration, mice brain sections were stained with 0.1% cresyl violet solution. Neurons were counted at 400× magnification in 4 separate non-overlapping fields. The number of neurons in the ipsilateral (right) side was normalized to those in the non-implanted contralateral (left) side [41].

### Statistical analysis

Data were tested by one-way analysis of variance (ANOVA) with Bonferroni *post hoc* tests or a two-tailed unpaired Student's *t* test. The level of significance was defined as *p*<0.05.

## Results

### HCV RNA quantification in infected brain and protein detection in infected microglia and astrocytes

**Figure 1:**
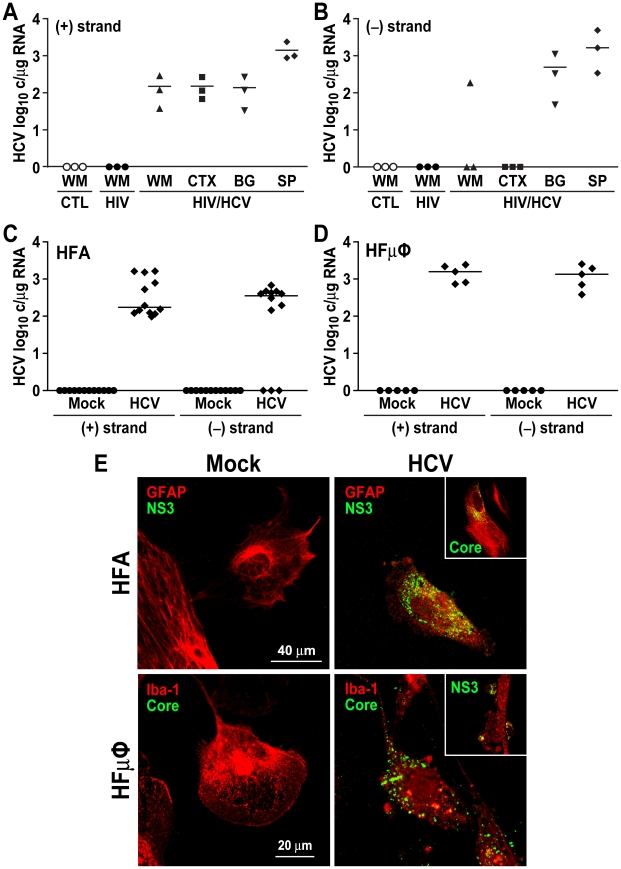
HCV RNA and proteins were detectable in different brain regions and infected glia cells. (A and B) Quantification of HCV-encoded positive- (A) and negative-strand (B) RNA of three brain regions from an HIV/HCV co-infected patient displayed different abundance of RNA in white matter (WM), cortex (CTX) and basal ganglion (BG) compared to spleen (SP). Negative-strand RNA of HCV was only detectable in basal ganglion, spleen and one sample from white matter. HCV RNA was not detected in white matter tissues from control (CTL) and HIV-1 mono-infected (HIV) individuals. (C and D) Primary cultures of human fetal astrocytes (HFA) and human fetal microglia (HFµφ) were exposed to supernatant containing HCV^cc^. The levels of HCV positive- and negative-strands were measured by real time RT-PCR with strand specific primers at day 3 post-infection (HFA, n = 12; HFµφ, n = 5). (E) Immunofluorescence of HCV core and NS3 proteins in J6/JFH1-infected primary HFA and HFµφ showed co-labeling of HCV-encoded proteins (green) with GFAP-immunoreactive astrocytes (red) or Iba-1-immunoreactive microglia (red) in HCV-infected cells at day 3 post-infection but not in mock-infected cells. Insets showed core and NS3-immunopositive HFA and HFµφ. (original magnification 630x)

HCV-encoded proteins as well as positive and negative RNA strands have been reported in different brain regions and identified specifically in microglia/macrophages and astrocytes [9], [10], [18]. To verify HCV infection of the brain occured, autopsied brain tissues were investigated in HIV/AIDS persons without HCV infection (n = 3) and an individual with HIV/AIDS and HCV infection at death. The latter individual was a 46-year old female patient with HIV-associated dementia (HIV Dementia Scale score  = 6; cranial MRI: cerebral atrophy with increased diffuse white matter signal on T2-weighted images), CD4^+^ T cell level  = 105 cells/µl and detectable HIV and HCV viremia. HCV RNA positive- and negative-strand copy numbers were determined in brain samples from all patients by a strand-specific reverse transcriptase with subsequent PCR amplification and interpreted from a standard curve. Positive-strand HCV RNA was detectable in white matter, cortex and basal ganglia from the HIV/HCV co-infected patient at 10^2^ viral copies/µg RNA, which were 10-fold lower than viral copy numbers in the matched spleen (Figure 1A). In contrast, negative-strand RNA, an indication of viral replication, was found only in white matter and basal ganglia but not in cortex (Figure 1B), as previously reported [10]. HCV-encoded RNA was not detected in control groups and HIV/AIDS persons without HCV viremia. To determine if human glia were permissive to HCV infection, primary cultures of HFAs and HFµφ were exposed to supernatants from J6/JFH1 transfected Huh 7.5 cells containing infectious virus (10^3^ FFU/ml, multiplicity of infection or m.o.i.  = 1). At day 3 post-infection, HCV positive-strand RNA were detected in all HCV^cc^-infected astrocytes at 10^2^ to 10^3^ viral copies/µg RNA while negative strands of HCV RNA were evident in only 75% of infected astrocyte cultures (Figure 1C). Similarly, HCV positive- and negative strands were measurable at 10^3^ viral copies/µg RNA in HCV^cc^-infected microglia (Figure 1D). Both HCV core and NS3 proteins were detectable in cytoplasm of GFAP (astrocytes) and Iba-1 (microglia) immunopositive cells compared to mock infection (Figure 1E and Figure S1). These studies highlighted the capacity of HCV to infect the select neural cells, particularly glial cells.

### HCV core protein induces differential inflammatory responses in microglia and astrocytes

**Figure 2:**
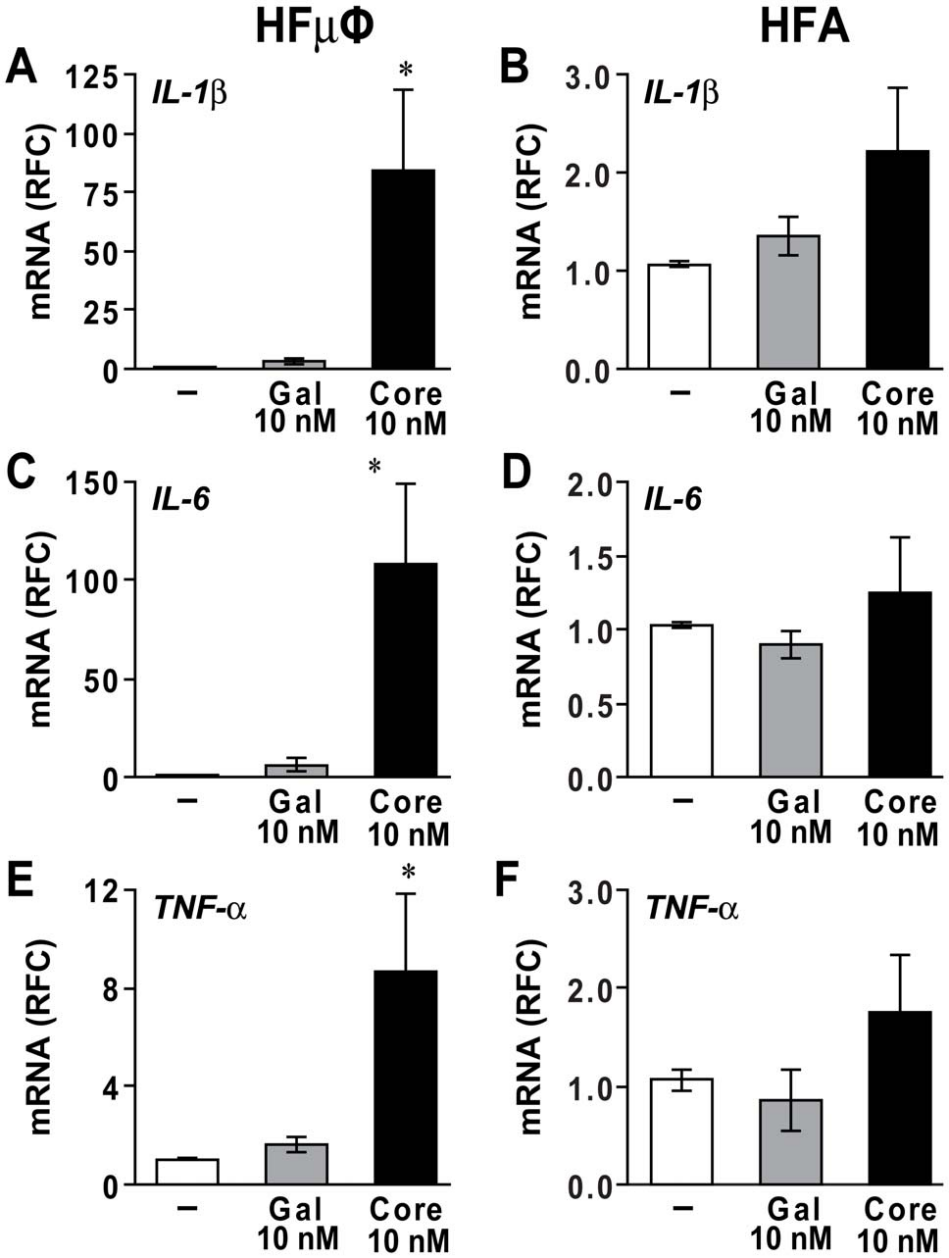
Recombinant HCV core protein activated microglia. Primary human fetal microglia (HFµφ) and astrocytes (HFA) were exposed to culture medium (mock, −), Gal (10 nM) or Gal-core (10 nM) for 12 hr. Gal-core-exposed HFµφ displayed elevated mRNA expression for *IL-1*β (A), *IL-6* (C) and *TNF-*α (E) while cytokine expression in Gal-exposed HFµφ was similar to mock-exposed cells. In contrast, Gal-core-exposed HFAs showed similar transcript levels for *IL-1*β (B), *IL-6* (D) and *TNF-*α (F) compared with mock-treated cells (n = 4). mRNA expression was reported as relative fold change (RFC) compared to *GAPDH* expression. Data represent mean±SEM for five or more independent experiments (one-way ANOVA with Bonferroni *post hoc* tests, * *p*<0.05 compared to mock-treated cells).

Exposure of HCV core protein to human blood monocytes activated the expression and release of pro-inflammatory cytokines [31]. To investigate the neuroinflammatory effects of HCV core on microglia/macrophages and astrocytes, HFµφ and HFAs were exposed to Gal-core or Gal (10 nM) for 12 hours and transcript levels of cytokines and chemokines were measured. HCV core induced expression of the pro-inflammatory cytokine *IL-1β* by 80 fold in HFµφ (Figure 2A, *p*<0.05) while there was only a trend of increased *IL-1β* expression in HFA (Figure 2B). Likewise, the expression of *IL-6* and *TNF-α* transcripts was significantly higher in HCV core-exposed HFµφ (Figure 2C and 2E, *p*<0.05) but not in HCV core-exposed HFAs (Figure 2D and 2F). Of interest, HCV core protein exposure did not activate the expression of interferon-alpha (*IFNα*) transcripts in both glial cell types (data not shown).

**Figure 3:**
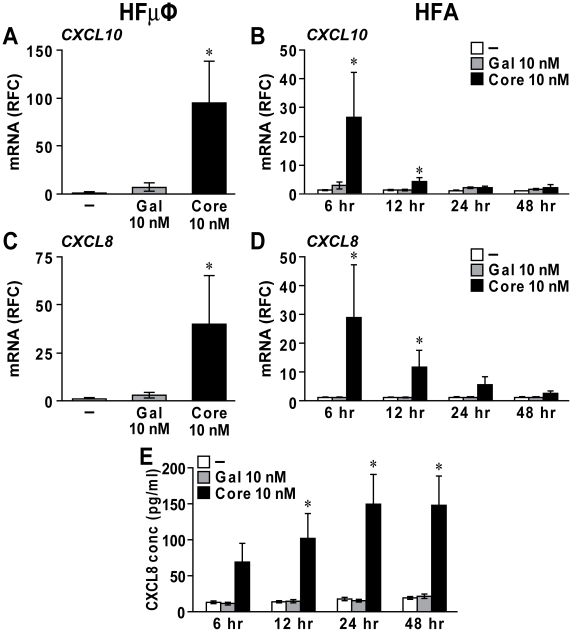
HCV core protein induced chemokine expression in microglia and astrocytes. Primary human fetal microglia (HFµφ) (A, C) and astrocytes (HFA) (B,D) exposed to 10 nM Gal-core displayed elevated mRNA expression for *CXCL10* (A, B) and *CXCL8* (C, D). The increased *CXCL10* and *CXCL8* expression in Gal-core-treated HFA was highest at 6 hr post-exposure and extinguished at 24 hr post-exposure. (E) Elevated levels of CXCL8 protein were detected in supernatants from Gal-core-treated HFAs after 12 hr exposure by ELISAs. Data represent mean±SEM for three or more independent experiments (one-way ANOVA with Bonferroni *post hoc* tests, * *p*<0.05 compared to mock-treated cells).

In addition to cytokine induction, HCV core protein has been reported to promote expression of chemokines, e.g. CXCL10, CXCL8 and CCL5, in monocytes and other cell types including hepatocytes and fibroblasts [31], [32], [43], [44]. After HCV core exposure (12 hr), HFµφ expressed elevated levels of *CXCL10* and *CXCL8* transcripts (Figure 3A and 3C, *p*<0.05). Similarly, transcript levels of *CXCL10* and *CXCL8* in HFAs were increased at 6 and 12 hr after HCV core protein application (Figure 3B and 3D, *p*<0.05). There was no difference in transcript levels for *CCL5* and *CXCL12* in HFAs (data not shown). The supernatant levels of CXCL8 protein derived from exposed HFAs increased with time and reached a plateau by 24 hr post-exposure (Figure 3E, *p*<0.05).

### Direct or indirect exposure of HCV core protein results in neuronal injury

**Figure 4:**
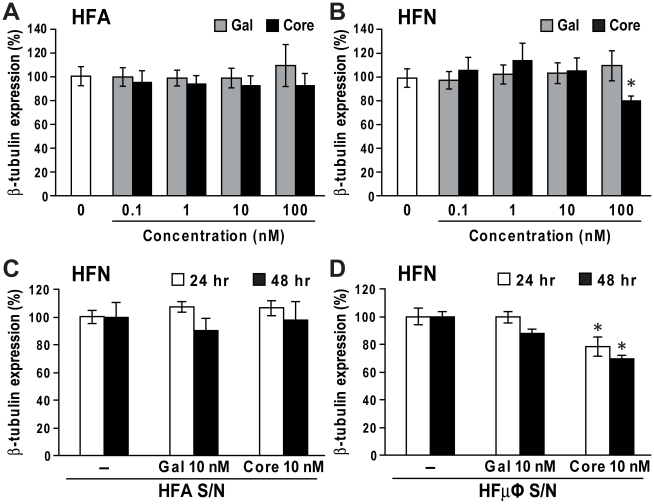
Neurotoxicity mediated by HCV core protein. Cell injury was determined by measuring β-III tubulin immunoreactivity at 48 hr post-exposure. (A) Gal or Gal-core exposure did not affect the viability of primary human fetal astrocytes (HFA). (B) Gal-core (100 nM) exposed to HFNs suppressed neuronal viability in terms of reduced β-tubulin immunoreactivity. (C) Supernatants from Gal- or Gal-core-treated astrocytes (HFA S/N) was not toxic to primary human fetal neurons (HFN, n = 3). (D) Supernatants from Gal-core-exposed microglia (HFµφ S/N) collected at 24 and 48 hr post-exposure also caused a reduction in β-tubulin immunoreactivity of HFNs. Data represent mean±SEM for three or more independent experiments (one-way ANOVA with Bonferroni *post hoc* tests, * *p*<0.05 compared to mock-treated cells).

Given HCV core protein has been detected in white and gray matters [9], its cytotoxic effects on HFAs and HFNs were investigated. HCV core protein exposure was not toxic to astrocytes (Figure 4A) while the highest concentration of HCV core protein (100 nM) was cytotoxic to neurons, indicated by the loss of β-III-tubulin expression compared to controls (Figure 4B, *p*<0.05). HCV core protein (100 nM) also reduced neuronal survival compared to controls as indicated by DAPI staining (data not shown). To determine if HCV core protein exerted its neurotoxic effects indirectly through induction of neurotoxic factors secreted by microglia or astrocytes, supernatants from HFAs or HFµφ exposed to Gal-core (10 nM) were collected at 24 and 48 hour post-exposure and then applied to HFNs. Supernatants from HCV core-exposed HFAs showed no neurotoxicity (Figure 4C) while supernatants from HCV core-exposed HFµφ caused neuronal injury (Figure 4D, *p*<0.05). These results suggested that HCV core protein was toxic to neurons through both direct and indirect mechanisms.

### HCV core protein alters neuronal membrane response and suppresses neuronal autophagy

**Figure 5:**
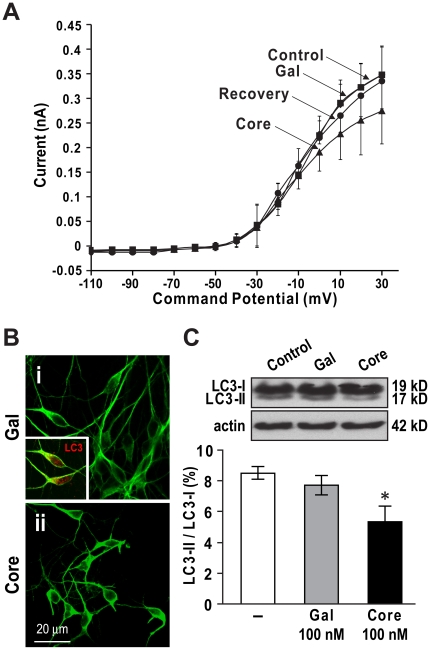
HCV core protein modulated neuronal membrane conductance. (A) Whole-cell currents recorded from human fetal neurons (HFNs) were reduced after exposure to Gal-core (100 nM, n = 4) but not Gal (100 nM, n = 6). (B) After Gal-core (100 nM) exposure, MAP-2-immunopositive HFNs (green) showed neurite retraction, fewer neurite processes, soma atrophy and loss of neurons (ii) compared to Gal (100 nM) exposure (i). The autophagy-related light chain 3 (LC3) molecule was widely expressed in unexposed neurons (red, i, inset) (original magnification 63×). (C) LC3 immunoreactivity of HFN lysates showed two isoforms of LC3: cytosolic LC3-I (top band) and its phosphatidylethanolamine-conjugated LC3-II (bottom band). 6 hr after exposure to 100 nM Gal-core, the band density ratio of LC3-II to LC3-I was reduced by 37.5% compared to mock-exposed cells. Data represent mean±SEM (n = 4, one-way ANOVA with Bonferroni *post hoc* tests, * *p*<0.05).

To define the mechanisms that underlie the direct neurotoxic effects of HCV core protein, the actions of HCV core protein were examined on neuronal membrane activity. Under voltage-clamp conditions in purified HFNs, HCV Gal-core (100 nM) reduced whole-cell outward currents in the voltage range from −30 to +30 mV relative to Gal exposure. This effect was reversible after the wash-out period (Figure 5A), indicating that HCV core protein was directly active at the neuronal membrane. Recently, neuronal autophagy suppression has been recognized as a key mechanism in several neurodegenerative disorders [45] and participates in the neurovirulence of several viruses [16], [46]. The role of autophagy in HCV core-mediated neurotoxicity was investigated by measuring the levels of light chain 3 (LC3), a marker of autophagosome formation. HFNs exposed to Gal protein displayed healthy MAP-2 immunopositive cells with long and complex neurites (Figure 5Bi); in contrast, HCV core-exposed neurons exhibited fewer MAP-2 immunopositive cells with shorter processes and atrophied soma (Figure 5Bii). Additionally, LC3 was highly expressed in Gal-exposed neurons (Figure 5Bi, inset). Western blot analysis of neuronal lysates showed a reduction of LC3-II expression in HCV core-exposed HFNs compared with control HFNs (Figure 5C, *p*<0.05), indicating that HCV core protein inhibited LC3-I to LC3-II conversion. These data implied that HCV core protein could act at the neuronal membrane, contributing to neuronal death by modulating neuronal autophagy.

### HIV-1 infection and Vpr protein potentiate the neuroimmune activation and neurotoxic effects of HCV core protein

**Figure 6:**
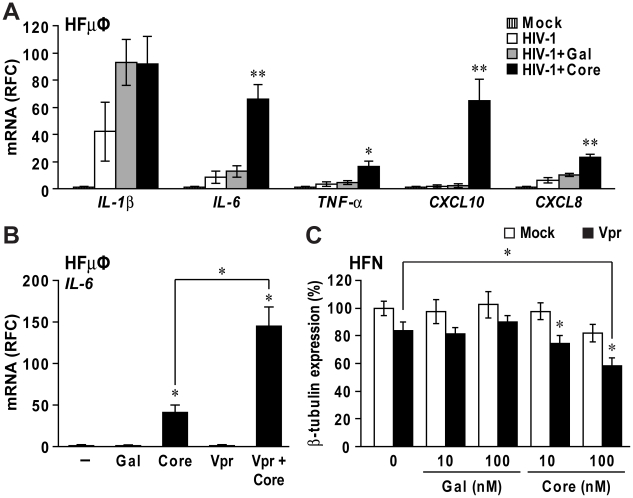
HCV core protein increased microglia activation and neurotoxicity induced by HIV-1 infection and Vpr protein. (A) Primary human fetal microglia (HFµφ) were infected with HIV-1 SF162. At day 2 post-infection, HIV-1-infected HFµφ were exposed to culture medium (HIV-1), Gal (10 nM, HIV-1+Gal) or Gal-core (10 nM, HIV-1+Core) for 12 hr. HIV-1 infection caused increased mRNA expression of *IL-1*β, *IL-6*, *TNF-*α and *CXCL8*. Exposure to Gal-core potentiated transcript expression of *IL-6*, *TNF-*α, *CXCL10* and *CXCL8* expression compared to HIV-1-infected microglia exposed to mock or Gal. (B) HFµφ exposed to HIV-1 Vpr (50 nM) and Gal-core (10 nM) proteins displayed elevated *IL-6* transcript levels compared to Vpr- or Gal-core-treated cells. (C) Concomitant exposure of primary human neurons (HFN) to HIV-1 Vpr and Gal-core also resulted in reduced β-tubulin immunoreactivity compared to mock- or Vpr-exposed cells in a concentration dependent manner. Data represent mean±SEM for three or more independent experiments (one-way ANOVA with Bonferroni *post hoc* tests, * *p*<0.05 and ** *p*<0.01 compared to mock-treated cells).

Brain mononuclear cells in HIV-infected persons are known to produce several proinflammatory cytokines such as IL-1β and TNF-α [14]. To determine if HCV core protein exacerbated cytokine and chemokine expression in HIV-1-infected microglia, HCV core protein was applied to HFµφ infected by HIV-1 SF162, a neurotropic HIV-1 strain. HIV-1 infection in microglia was confirmed by detecting HIV-1 *pol* expression (data not shown). As expected, HIV-1 infection induced expression of *IL-1β*, *IL-6*, *TNF-α* and *CXCL8* (Figure 6A). However, HCV core exposure (12 hr) increased *IL-6*, *TNF-α*, *CXCL10* and *CXCL8* expression from 2 to 29 fold compared to Gal-exposed cells (Figure 6A, *p*<0.05). These studies were extended by showing that concurrent exposure of HIV-1 Vpr with HCV core proteins caused higher *IL-6* expression than HCV core-treated HFµφ (Figure 6B) while there was no additive effect on the induction of other cytokines and chemokines (data not shown). In addition, soluble HIV-1 Vpr protein at a subneurotoxic concentration (50 nM) together with HCV core protein (10 and 100 nM) caused significantly increased HFN injury (Figure 6C) indicated by reduced β-III-tubulin immunodetection, compared with HFNs exposed to either HIV-1 Vpr or HCV core proteins at the same concentrations (*p*<0.05). These results indicated that concurrent exposure of HIV-1 and HCV encoded proteins potentiated the neuroimmune activation and neurotoxicity effects of HCV core protein.

### HCV core protein causes neuropathological and neurobehavioral deficits in Vpr-transgenic mice

**Figure 7:**
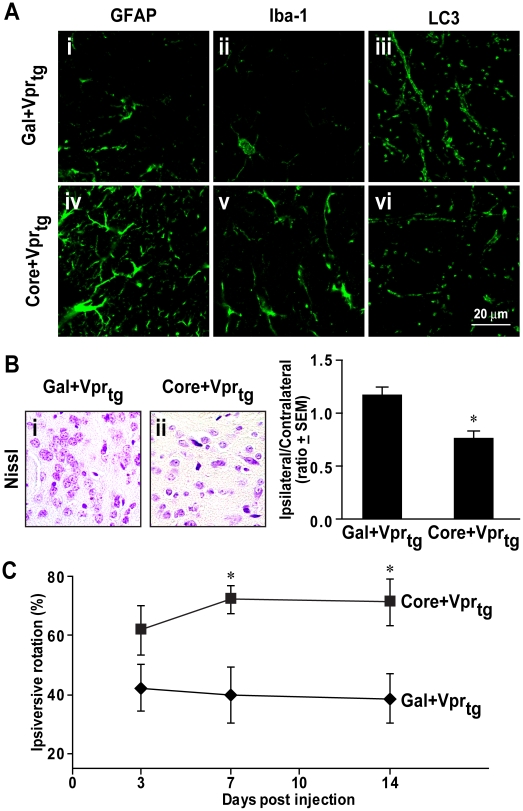
Implanted HCV core protein caused glial activation, neuronal loss and neurobehavioral deficits in Vpr-transgenic mice. (A) GFAP and Iba-1 immunoreactivities were minimally detected in ipsilateral brain hemispheres of animals implanted with Gal (i, ii) while higher numbers of GFAP and Iba-1 immunopositive cells were evident in Gal-core-implanted animals (iv, v). LC3 immunoreactivity was lower in Gal-core-implanted animals (vi) compared to Gal-implanted animals (iii) (original magnification 63×). (B) Nissl staining of ipsilateral basal ganglia displayed lower numbers of neurons in Vpr transgenic mice implanted with Gal-core (ii) compared with Gal (i) (original magnification 400×). Neurons were counted in four different fields of four sections from ipsilateral and contralateral hemispheres. Gal-core-implanted Vpr transgenic mice displayed a lower ratio of ipsilateral to contralateral neuronal counts (n = 3, two-tailed unpaired Student's *t* test, * *p*<0.05). (C) *In vivo* neurological damage in Gal-core-implanted Vpr transgenic mice was indicated by higher frequencies of ipsiversive rotations at days 7 and 14 post-implantation (n = 6, repeated measured ANOVA with Bonferroni *post hoc* tests, * *p*<0.05 compared to Gal-implanted animals). Data are presented as the mean±SEM.

As the present *in vitro* studies exhibited additive neurotoxic effects of HCV core and HIV-1 Vpr proteins, Gal-core or Gal proteins were stereotactically implanted into the right striatum of HIV-1 Vpr-transgenic mice. These transgenic animals were previously shown to exhibit features indicative of neurodegeneration [15], [47]. Morphological analysis at day 14 post-implantation disclosed increased immunoreactivity of GFAP, an astrocyte marker, and Iba-1, a microglia/macrophage marker, in the right striatum of Vpr-transgenic mice receiving HCV core protein implantation (Figure 7Aiv and v). Additionally, HCV core-implanted animals displayed lower LC3 immunoreactivity (Figure 7Avi) and reduced numbers of cresyl violet-positive cells in the right striatum (Figure 7Bii). Mean ratios of ipsilateral to contralateral neuronal counts confirmed neuronal loss in the implanted striatum (Figure 7B). In conjunction with these latter neuropathological features, Vpr transgenic mice receiving HCV core implantation exhibited greater ipsiversive rotation frequency at days 7 and 14 post-implantation (Figure 7C, *p*<0.05). Taken together, the *in vivo* neurotoxicity of HCV core protein was likely a consequence of autophagy suppression in neurons coupled with activation of proximate astrocytes and microglia leading to neuronal injury and loss.

## Discussion

During the last decade, there is mounting evidence to suggest HCV is neuroinvasive and HIV/HCV co-infected patients display higher rates of neuropsychological deficits. To delineate the underlying pathogenic mechanisms of HCV infection of the brain, we report for the first time that primary human astrocytes and microglia were permissive to HCV infection with cell culture-derived HCV particles. Moreover, this report demonstrates for the first time that HCV core protein activates human glia and contributes to neurotoxicity. Direct exposure of HCV core protein to primary human neurons suppressed the neuronal autophagy, leading to neurite retraction. The change in neuronal membrane potential after exposure to HCV core protein indicated that core was biologically active at the cell membrane and was able to modulate ionic conductance in neurons. In addition to direct neurotoxicity, proinflammatory cytokines and other neurotoxins released from HCV core-activated microglia into supernatants were toxic to neurons. The *in vitro* and *in vivo* aberrant immune activation and neurotoxicity mediated by HCV core protein were amplified in the presence of HIV-1 Vpr protein. These findings support the concept that the presence of HCV-encoded proteins cause neuronal damage and perhaps neurocognitive impairment in individuals co-infected with HIV and HCV.

Although HCV tropism is principally recognized in human liver cells, extrahepatic replication has been reported in PBMC [48], myocardium [49], [50] and brain cells [10], [18] by detecting HCV negative-strand RNA. Herein, detection of positive- and negative-strand RNA in different brain regions was performed in a patient with HIV and HCV co-infection and was consistent with previous reports [10], [18]. HCV^cc^ infects and replicates in Huh 7.5 hepatocytes. However, productive infection was not detected in other hepatic cell lines (e.g. HepG2) [20], non-hepatic cell lines (e.g. HeLa, 293T and U-937) [20] and PBMC (e.g. B and T lymphocytes, monocytes and dendritic cells) [51], likely due to the lack of some entry receptors e.g. claudin-1 and their partner proteins [51] or host factors required for HCV infection e.g. liver specific microRNA miR-122 [52], [53], [54]. Herein, expression of HCV proteins, core and NS3, were immunodetected in the cytoplasm of primary human astrocytes and microglia infected with HCV^cc^. Although the levels of infectivity in primary astrocytes and microglia were low (less than 1%), HCV positive- and negative-strand RNA were measurable in infected astrocytes, suggesting that HCV is capable of replication in astrocytes. Both microglia and astrocytes were previously identified as HCV-infected cells in autopsied brains [9], [18] and were shown to express SR-BI, CD81, claudin-1 and occludin [22], [23], [24], [25], [26]. Although the expression levels of claudin-1 and occludin were 15 to 50 fold lower in primary human astrocytes and microglia compared with Huh 7.5 cells (Figure S2A), claudin-1 expression in glial cells was approximately 10 fold higher than in PBMC or 293T cells [51]. The higher expression of HCV entry receptors in primary astrocytes compared to microglia was also consistent with less frequently-detected HCV-immunopositive microglia after HCV^cc^ infection. HCV replication in infected or transfected hepatocytes was reported in the membranous web structure closely associated with rough endoplasmic reticulum [55]. In the present studies, localization of HCV proteins in astrocytes was similarly associated with structures resembling endoplasmic reticulum. Further studies to determine if HCV-infected microglia or astrocytes release infectious viruses will be of interest.

HCV^cc^ used herein belongs to the HCV genotype 2a [19] while most HCV strains in North America are genotypes 1a or 1b, which are associated with higher HCV RNA levels, poor responses to IFN-α treatment and lower mean CD4 count in HIV/HCV co-infected individuals [2]. The strain difference and other co-morbidities such as intravenous drug use might influence the infection and production of HCV in human brain, as demonstrated for HIV infection [56].

HCV infection has been correlated with cognitive impairment with changes indicative of neuronal damage by magnetic resonance spectroscopy [8]. Herein, we demonstrated that the direct exposure of HCV core protein or exposure to supernatants from HCV core-exposed microglia were toxic to primary human neurons together with neurobehavioral deficits and neuronal loss in HCV core-implanted animals. Although the toxic concentration of HCV core for primary human neurons in our *in vitro* experiments was higher than the reported serum levels in HCV-infected patients, it was consistent with previous studies [31], [32], [57]. It is plausible that HCV core concentrations at the surface of infected and proximate (target) cells are higher than in serum; moreover, chronic and repeated HCV exposures might yield augmented neurotoxic effects. Remarkably, the concentration of HCV core required to activate microglia and astrocytes was one log less than the concentration needed for direct neurotoxicity.

HIV-1 Vpr is highly neurotoxic without substantial proinflammatory properties [58] and its expression in brain monocytic cells contributes to synaptic injury and neurobehavioral abnormalities in transgenic mice [15]. At subtoxic concentrations, simultaneous exposure of HCV core and HIV-1 Vpr proteins to neurons exerted additive effects, resulting in increased IL-6 expression in glial cells and neuronal injury. Likewise, implantation of HCV core into the striatum of HIV-1 Vpr transgenic mice resulted in marked glial activation coupled with neuronal loss. It is likely that Vpr and HCV core act through different mechanisms to yield cumulative neurotoxic effects as Vpr's putative receptor is the nuclear glucocorticoid receptor [59] while HCV core's receptor might be a cell membrane protein [31], [32], [57], [60]. Additionally, it is conceivable that HCV core protein might also potentiate the neurotoxic or neuroinflammatory effects of other HIV-1 proteins e.g. Tat, Nef or gp120. This possibility might highlight the importance of interactions between HCV and HIV and warrants further investigation.

Our data showed that core protein from HCV genotype 1b triggered *in vitro* and *in vivo* activation of microglia and astrocytes, which is consistent with a recent magnetic resonance spectroscopy study that revealed an increased level of *myo*-inositol, a hallmark of gliosis, in frontal white matter of HCV-infected individuals [61]. Increased serum TNF-α and IL-1β levels in HCV-infected individuals were correlated with severity of neuropsychiatric dysfunction [62]. *In vitro* HCV infection in primary human macrophages resulted in increased TNF-α and CXCL8 [63]. Increased expression of CXCL10 and CXCL8 in astrocytes was also reported in brains of individuals with HIV encephalitis [64]. A recent study demonstrated that HIV-infected macrophages induced CXCL8 production from astrocytes through IL-1β and TNF-α [65]. The chemoattractant CXCL10 plays an important role in leukocyte infiltration into various tissues including CNS [66]. CXCL8 recruits not only neutrophils but also monocytes and lymphocytes [67]. It is plausible that the increased expression of CXCL10 and CXCL8 by HCV core-exposed microglia and astrocytes might enhance recruitment of T helper cells and monocytes in the brain, leading to deleterious effects. Of interest, we also observed that HCV core exposure to microglia induced the transcript levels of indoleamine 2,3-dioxygenase (IDO) (data not shown), which has been linked to neuronal injury [68], [69]. Additionally, we also demonstrated that supernatants from HCV core-treated microglia were neurotoxic. In these experiments, the concentration of HCV core protein was subtoxic to neurons but was sufficient to induce the expression of neurotoxic factors. It is also possible that there was carry-over of HCV core protein in supernatants which might play a role in the overall neurotoxicity. However, these results indicate that exposure of HCV core protein to glial cells could lead to deleterious effects on neurons.

Several studies have reported that recombinant HCV core activated cell surface receptors e.g. Toll-like receptor 2 (TLR2) on monocytes [31] and dendritic cells [70] or the putative HCV core receptor, gC1qR, on T lymphocytes [57], [60] and lung fibroblasts [32]. Microglia/macrophages are known to express both receptors [71], [72] while astrocytes express gC1qR but not TLR2 [72], [73] and there was a 30% increase in the gC1qR transcript level in HIV-infected brains (Figure S2B). Inhibition of either TLR2 or gC1qR by blocking antibodies or RNA silencing will be of interest. Nonetheless, HCV core might also be endocytosed by the microglia or astrocytes, and subsequently act through intracellular or nuclear receptors.

In summary, we reported primary human microglia and astrocytes were permissive to HCV infection and HCV-encoded protein, core, was neurotoxic but also activated pro-inflammatory responses in human microglia and astrocytes. The augmented glial activation and neurotoxicity mediated by HCV core protein in the presence of HIV-1 Vpr protein or HIV-1 infection highlighted the additive effects of HCV- and HIV-encoded proteins in the pathogenesis of neurologic disease. Future studies are required to delineate the precise mechanisms by which HIV and HCV proteins interact and amplify neuropathogenesis, thereby permitting the development of potential management and therapeutic strategies.

## Supporting Information

Table S1 Real-time RT PCR primer sequences for detecting human genes.
(0.06 MB DOC)

Figure S1 Primary human astrocytes and microglia were permissive to infection by HCVcc. At day 3 post-infection, HCV core protein immunoreactivity (green) was colocalized with GFAP or Iba-1 (red) in HCVcc-infected primary human fetal astrocytes (HFA) and microglia (HFµΦ) but not in mock-infected cells. (original magnification 630x)
(4.39 MB TIF)

Figure S2 Expression of HCV entry receptors in glial cells and brains. (A) Transcript expression of HCV entry receptors were determined by real time RT-PCR. Primary human microglia and astrocytes expressed all known HCV entry receptors, albeit at the lower levels than Huh 7.5 hepatoma cell line. (B) Similar levels of HCV entry receptors were found in white matter tissues from control and individuals with HIV encephalitis (HIV) while the expression of the *gC1qR* transcript was elevated in HIV compared to control (n = 5, * p<0.05). Data represent mean ±SEM for three or more independent experiments.
(0.81 MB TIF)

## References

[1] Kim AY, Chung RT (2009). Coinfection with HIV-1 and HCV–a one-two punch.. Gastroenterology.

[2] Jones R, Dunning J, Nelson M (2005). HIV and hepatitis C co-infection.. Int J Clin Pract.

[3] Greub G, Ledergerber B, Battegay M, Grob P, Perrin L (2000). Clinical progression, survival, and immune recovery during antiretroviral therapy in patients with HIV-1 and hepatitis C virus coinfection: the Swiss HIV Cohort Study.. Lancet.

[4] Laskus T, Radkowski M, Adair DM, Wilkinson J, Scheck AC (2005). Emerging evidence of hepatitis C virus neuroinvasion.. Aids.

[5] Clifford DB, Yang Y, Evans S (2005). Neurologic consequences of hepatitis C and human immunodeficiency virus coinfection.. J Neurovirol.

[6] Hilsabeck RC, Castellon SA, Hinkin CH (2005). Neuropsychological aspects of coinfection with HIV and hepatitis C virus.. Clin Infect Dis.

[7] Ryan EL, Morgello S, Isaacs K, Naseer M, Gerits P (2004). Neuropsychiatric impact of hepatitis C on advanced HIV.. Neurology.

[8] Forton DM, Allsop JM, Cox IJ, Hamilton G, Wesnes K (2005). A review of cognitive impairment and cerebral metabolite abnormalities in patients with hepatitis C infection.. Aids.

[9] Letendre S, Paulino AD, Rockenstein E, Adame A, Crews L (2007). Pathogenesis of hepatitis C virus coinfection in the brains of patients infected with HIV.. J Infect Dis.

[10] Radkowski M, Wilkinson J, Nowicki M, Adair D, Vargas H (2002). Search for hepatitis C virus negative-strand RNA sequences and analysis of viral sequences in the central nervous system: evidence of replication.. J Virol.

[11] Murray J, Fishman SL, Ryan E, Eng FJ, Walewski JL (2008). Clinicopathologic correlates of hepatitis C virus in brain: a pilot study.. J Neurovirol.

[12] Laskus T, Radkowski M, Bednarska A, Wilkinson J, Adair D (2002). Detection and analysis of hepatitis C virus sequences in cerebrospinal fluid.. J Virol.

[13] Fishman SL, Murray JM, Eng FJ, Walewski JL, Morgello S (2008). Molecular and bioinformatic evidence of hepatitis C virus evolution in brain.. J Infect Dis.

[14] Jones G, Power C (2006). Regulation of neural cell survival by HIV-1 infection.. Neurobiol Dis.

[15] Jones GJ, Barsby NL, Cohen EA, Holden J, Harris K (2007). HIV-1 Vpr causes neuronal apoptosis and in vivo neurodegeneration.. J Neurosci.

[16] Zhu Y, Vergote D, Pardo C, Noorbakhsh F, McArthur JC (2009). CXCR3 activation by lentivirus infection suppresses neuronal autophagy: neuroprotective effects of antiretroviral therapy.. Faseb J.

[17] Alirezaei M, Kiosses WB, Flynn CT, Brady NR, Fox HS (2008). Disruption of neuronal autophagy by infected microglia results in neurodegeneration.. PLoS One.

[18] Wilkinson J, Radkowski M, Laskus T (2009). Hepatitis C virus neuroinvasion: identification of infected cells.. J Virol.

[19] Lindenbach BD, Evans MJ, Syder AJ, Wolk B, Tellinghuisen TL (2005). Complete replication of hepatitis C virus in cell culture.. Science.

[20] Zhong J, Gastaminza P, Cheng G, Kapadia S, Kato T (2005). Robust hepatitis C virus infection in vitro.. Proc Natl Acad Sci U S A.

[21] Burlone ME, Budkowska A (2009). Hepatitis C virus cell entry: role of lipoproteins and cellular receptors.. J Gen Virol.

[22] Husemann J, Loike JD, Anankov R, Febbraio M, Silverstein SC (2002). Scavenger receptors in neurobiology and neuropathology: their role on microglia and other cells of the nervous system.. Glia.

[23] Husemann J, Silverstein SC (2001). Expression of scavenger receptor class B, type I, by astrocytes and vascular smooth muscle cells in normal adult mouse and human brain and in Alzheimer's disease brain.. Am J Pathol.

[24] Dijkstra S, Geisert EJ, Gispen WH, Bar PR, Joosten EA (2000). Up-regulation of CD81 (target of the antiproliferative antibody; TAPA) by reactive microglia and astrocytes after spinal cord injury in the rat.. J Comp Neurol.

[25] Duffy HS, John GR, Lee SC, Brosnan CF, Spray DC (2000). Reciprocal regulation of the junctional proteins claudin-1 and connexin43 by interleukin-1beta in primary human fetal astrocytes.. J Neurosci.

[26] Romanitan MO, Popescu BO, Winblad B, Bajenaru OA, Bogdanovic N (2007). Occludin is overexpressed in Alzheimer's disease and vascular dementia.. J Cell Mol Med.

[27] Maillard P, Krawczynski K, Nitkiewicz J, Bronnert C, Sidorkiewicz M (2001). Nonenveloped nucleocapsids of hepatitis C virus in the serum of infected patients.. J Virol.

[28] Soffredini R, Rumi MG, Parravicini ML, Ronchi G, Del Ninno E (2004). Serum levels of hepatitis C virus core antigen as a marker of infection and response to therapy.. Am J Gastroenterol.

[29] Fischer R, Baumert T, Blum HE (2007). Hepatitis C virus infection and apoptosis.. World J Gastroenterol.

[30] Giannini C, Brechot C (2003). Hepatitis C virus biology.. Cell Death Differ.

[31] Dolganiuc A, Oak S, Kodys K, Golenbock DT, Finberg RW (2004). Hepatitis C core and nonstructural 3 proteins trigger toll-like receptor 2-mediated pathways and inflammatory activation.. Gastroenterology.

[32] Moorman JP, Fitzgerald SM, Prayther DC, Lee SA, Chi DS (2005). Induction of p38- and gC1qR-dependent IL-8 expression in pulmonary fibroblasts by soluble hepatitis C core protein.. Respir Res.

[33] Eisen-Vandervelde AL, Yao ZQ, Hahn YS (2004). The molecular basis of HCV-mediated immune dysregulation.. Clin Immunol.

[34] Power C, McArthur JC, Nath A, Wehrly K, Mayne M (1998). Neuronal death induced by brain-derived human immunodeficiency virus type 1 envelope genes differs between demented and nondemented AIDS patients.. J Virol.

[35] Vecil GG, Larsen PH, Corley SM, Herx LM, Besson A (2000). Interleukin-1 is a key regulator of matrix metalloproteinase-9 expression in human neurons in culture and following mouse brain trauma in vivo.. J Neurosci Res.

[36] Johnston JB, Jiang Y, van Marle G, Mayne MB, Ni W (2000). Lentivirus infection in the brain induces matrix metalloproteinase expression: role of envelope diversity.. J Virol.

[37] Wakita T, Pietschmann T, Kato T, Date T, Miyamoto M (2005). Production of infectious hepatitis C virus in tissue culture from a cloned viral genome.. Nat Med.

[38] Power C, McArthur JC, Johnson RT, Griffin DE, Glass JD (1995). Distinct HIV-1 env sequences are associated with neurotropism and neurovirulence.. Curr Top Microbiol Immunol.

[39] Yuki N, Matsumoto S, Tadokoro K, Mochizuki K, Kato M (2006). Significance of liver negative-strand HCV RNA quantitation in chronic hepatitis C.. J Hepatol.

[40] Dickie P, Roberts A, Uwiera R, Witmer J, Sharma K (2004). Focal glomerulosclerosis in proviral and c-fms transgenic mice links Vpr expression to HIV-associated nephropathy.. Virology.

[41] Johnston JB, Zhang K, Silva C, Shalinsky DR, Conant K (2001). HIV-1 Tat neurotoxicity is prevented by matrix metalloproteinase inhibitors.. Ann Neurol.

[42] Ungerstedt U, Arbuthnott GW (1970). Quantitative recording of rotational behavior in rats after 6-hydroxy-dopamine lesions of the nigrostriatal dopamine system.. Brain Res.

[43] Ruggieri A, Franco M, Gatto I, Kumar A, Rapicetta M (2007). Modulation of RANTES expression by HCV core protein in liver derived cell lines.. BMC Gastroenterol.

[44] Apolinario A, Majano PL, Lorente R, Nunez O, Clemente G (2005). Gene expression profile of T-cell-specific chemokines in human hepatocyte-derived cells: evidence for a synergistic inducer effect of cytokines and hepatitis C virus proteins.. J Viral Hepat.

[45] Martinez-Vicente M, Cuervo AM (2007). Autophagy and neurodegeneration: when the cleaning crew goes on strike.. Lancet Neurol.

[46] Orvedahl A, Levine B (2008). Autophagy and viral neurovirulence.. Cell Microbiol.

[47] Noorbakhsh F, Ramachandran R, Barsby N, Ellestad KK, Leblanc A MicroRNA profiling reveals new aspects of HIV neurodegeneration: caspase-6 regulates astrocyte survival.. Faseb J.

[48] Zignego AL, Giannini C, Monti M, Gragnani L (2007). Hepatitis C virus lymphotropism: lessons from a decade of studies.. Dig Liver Dis.

[49] Sanchez MJ, Bergasa NV (2008). Hepatitis C associated cardiomyopathy: potential pathogenic mechanisms and clinical implications.. Med Sci Monit.

[50] Boddi M, Abbate R, Chellini B, Giusti B, Giannini C Hepatitis C virus RNA localization in human carotid plaques.. J Clin Virol.

[51] Marukian S, Jones CT, Andrus L, Evans MJ, Ritola KD (2008). Cell culture-produced hepatitis C virus does not infect peripheral blood mononuclear cells.. Hepatology.

[52] Bode JG, Brenndorfer ED, Haussinger D (2008). Hepatitis C virus (HCV) employs multiple strategies to subvert the host innate antiviral response.. Biol Chem.

[53] Moriishi K, Matsuura Y (2007). Host factors involved in the replication of hepatitis C virus.. Rev Med Virol.

[54] Randall G, Panis M, Cooper JD, Tellinghuisen TL, Sukhodolets KE (2007). Cellular cofactors affecting hepatitis C virus infection and replication.. Proc Natl Acad Sci U S A.

[55] Moradpour D, Gosert R, Egger D, Penin F, Blum HE (2003). Membrane association of hepatitis C virus nonstructural proteins and identification of the membrane alteration that harbors the viral replication complex.. Antiviral Res.

[56] Anthony IC, Arango JC, Stephens B, Simmonds P, Bell JE (2008). The effects of illicit drugs on the HIV infected brain.. Front Biosci.

[57] Yao ZQ, Nguyen DT, Hiotellis AI, Hahn YS (2001). Hepatitis C virus core protein inhibits human T lymphocyte responses by a complement-dependent regulatory pathway.. J Immunol.

[58] Piller SC, Jans P, Gage PW, Jans DA (1998). Extracellular HIV-1 virus protein R causes a large inward current and cell death in cultured hippocampal neurons: implications for AIDS pathology.. Proc Natl Acad Sci U S A.

[59] Refaeli Y, Levy DN, Weiner DB (1995). The glucocorticoid receptor type II complex is a target of the HIV-1 vpr gene product.. Proc Natl Acad Sci U S A.

[60] Kittlesen DJ, Chianese-Bullock KA, Yao ZQ, Braciale TJ, Hahn YS (2000). Interaction between complement receptor gC1qR and hepatitis C virus core protein inhibits T-lymphocyte proliferation.. J Clin Invest.

[61] Forton DM, Hamilton G, Allsop JM, Grover VP, Wesnes K (2008). Cerebral immune activation in chronic hepatitis C infection: a magnetic resonance spectroscopy study.. J Hepatol.

[62] Loftis JM, Huckans M, Ruimy S, Hinrichs DJ, Hauser P (2008). Depressive symptoms in patients with chronic hepatitis C are correlated with elevated plasma levels of interleukin-1beta and tumor necrosis factor-alpha.. Neurosci Lett.

[63] Radkowski M, Bednarska A, Horban A, Stanczak J, Wilkinson J (2004). Infection of primary human macrophages with hepatitis C virus in vitro: induction of tumour necrosis factor-alpha and interleukin 8.. J Gen Virol.

[64] Sanders VJ, Pittman CA, White MG, Wang G, Wiley CA (1998). Chemokines and receptors in HIV encephalitis.. Aids.

[65] Zheng JC, Huang Y, Tang K, Cui M, Niemann D (2008). HIV-1-infected and/or immune-activated macrophages regulate astrocyte CXCL8 production through IL-1beta and TNF-alpha: involvement of mitogen-activated protein kinases and protein kinase R.. J Neuroimmunol.

[66] Klein RS (2004). Regulation of neuroinflammation: the role of CXCL10 in lymphocyte infiltration during autoimmune encephalomyelitis.. J Cell Biochem.

[67] Mukaida N (2000). Interleukin-8: an expanding universe beyond neutrophil chemotaxis and activation.. Int J Hematol.

[68] Afkhami-Goli A, Liu SH, Zhu Y, Antony JM, Arab H (2009). Dual lentivirus infection potentiates neuroinflammation and neurodegeneration: viral copassage enhances neurovirulence.. J Neurovirol.

[69] Pais TF, Figueiredo C, Peixoto R, Braz MH, Chatterjee S (2008). Necrotic neurons enhance microglial neurotoxicity through induction of glutaminase by a MyD88-dependent pathway.. J Neuroinflammation.

[70] Dolganiuc A, Kodys K, Kopasz A, Marshall C, Do T (2003). Hepatitis C virus core and nonstructural protein 3 proteins induce pro- and anti-inflammatory cytokines and inhibit dendritic cell differentiation.. J Immunol.

[71] Barbasz A, Guevara-Lora I, Rapala-Kozik M, Kozik A (2008). Kininogen binding to the surfaces of macrophages.. Int Immunopharmacol.

[72] Jack CS, Arbour N, Manusow J, Montgrain V, Blain M (2005). TLR signaling tailors innate immune responses in human microglia and astrocytes.. J Immunol.

[73] Fernando LP, Natesan S, Joseph K, Kaplan AP (2003). High molecular weight kininogen and factor XII binding to endothelial cells and astrocytes.. Thromb Haemost.

