# Supplementary material for: Hepatitis C Virus Core Protein Induces Neuroimmune Activation and Potentiates Human Immunodeficiency Virus-1 Neurotoxicity
Source: PLoS One. 2010 Sep 21;5(9):e12856. doi: 10.1371/journal.pone.0012856 (PMC2943470; doi:10.1371/journal.pone.0012856)
Supplement: Table S1 — Real-time RT PCR primer sequences for detecting human genes. (0.06 MB DOC) [file pone.0012856.s001.doc]

**Table S1. Real-time RT PCR primer sequences for detecting human genes.**

| Primer name | Sequence | Reference |
| --- | --- | --- |
| *GAPDH* forward | 5’-AGC CTT CTC CAT GGT GGT GAA GAC-3’ | Noorbakhsh et al. J Exp Med. |
| *GAPDH* reverse | 5’-CGG AGT CAA CGG ATT TGG TCG-3’ | 2006 February 20; 203(2): 425–35. |
| *IL-1*β forward | 5’-CCA AAG AAG AAG ATG GAA AAG C-3’ | Jones et al. Virology. |
| *IL-1*β reverse | 5’-GGT GCT GAT GTA CCA GTT GGG-3’ | 2005 April 10; 334(2): 178-93. |
| *IL-6* forward | 5’-ACC CCT GAC CCA ACC ACA AAT-3’ | Jones et al. Virology. |
| *IL-6* reverse | 5’-AGC TGC GCA GAA TGA GAT GAG-3’ | 2005 April 10; 334(2): 178-93. |
| *TNF*α forward | 5’-CCC AGG GAC CTC TCT CTA ATC A-3’ | Real time primer database assay #2704, |
| *TNF*α reverse | 5’-GCT ACA GGC TTG TCA CTC GG-3’ | <http://medgen.ugent.be/RTPrimerDB> |
| *IFN*α forward | 5’-GGA GGA GAG GGT GGG AGA AAC-3’ | - |
| *IFN*α reverse | 5’-GAA AGC GTG ACC TGG TGT ATG AG-3’ |  |
| *CXCL10* forward | 5’-TCG AAG GCC ATC AAG AAT TT-3’ | Tang et al. Clin Chem. 2005 December; |
| *CXCL10* reverse | 5’-GCT CCC CTC TGG TTT TAA GG-3’ | 51(12): 2333-40. |
| *IL-8* forward | 5’-TTC AGC TCT GCA TCG TTT TG-3’ | - |
| *IL-8* reverse | 5’-CAC CGG AAG GAA CCA TCT CAC-3’ |  |
| *MIG* forward | 5’-GAG TGC AAG GAA CCC CAG TAG T-3’ | Real time primer database assay #1791, |
| *MIG* reverse | 5’-TTG TAG GTG GAT AGT CCC TTG GT-3’ | <http://medgen.ugent.be/RTPrimerDB> |
| *IDO* forward | 5’-GGC AAA CTG GAA GAA AAA AGG-3’ | Noorbakhsh et al. Virology. |
| *IDO* reverse | 5’-ATT TCC ACC AAT AGA GAG AC-3’ | 2006 May 10; 348 (2): 260-76 |
| *SR-BI* forward | 5’-CTG TGG GTG AGA TCA TGT GG-3’ | Sporstøl et al., BMC Mol Biol. 2007 |
| *SR-BI* reverse | 5’-GCC AGA AGT CAA CCT TGC TC-3’ | Jan 22; 8: 5. |
| *CD81* forward | 5’-CGC CAA GGA TGT GAA GCA GTT C-3’ | Kronenberger et al., Hepatology. 2001 |
| *CD81* reverse | 5’-TCC CGG AGA AGA GGT CAT CGA T-3’ | Jun; 33(6): 1518-26. |
| *CLDN1* forward | 5’-CCT ATG ACC CCA GTC AAT GC-3’ | Real time primer database assay #3761, |
| *CLDN1* reverse | 5’-TCC CAG AAG GCA GAG AGA AG-3’ | <http://medgen.ugent.be/RTPrimerDB> |
| *gC1qR* forward | 5’-TAA CAA CAG CAT CCC ACC AA-3’ | - |
| *gC1qR* reverse | 5’-GCC TTC TTG CCA TCA TCA TT-3’ |  |
