# Supplementary figures and images for: Hepatitis C Virus Core Protein Induces Neuroimmune Activation and Potentiates Human Immunodeficiency Virus-1 Neurotoxicity
Source: PLoS One. 2010 Sep 21;5(9):e12856. doi: 10.1371/journal.pone.0012856 (PMC2943470; doi:10.1371/journal.pone.0012856)

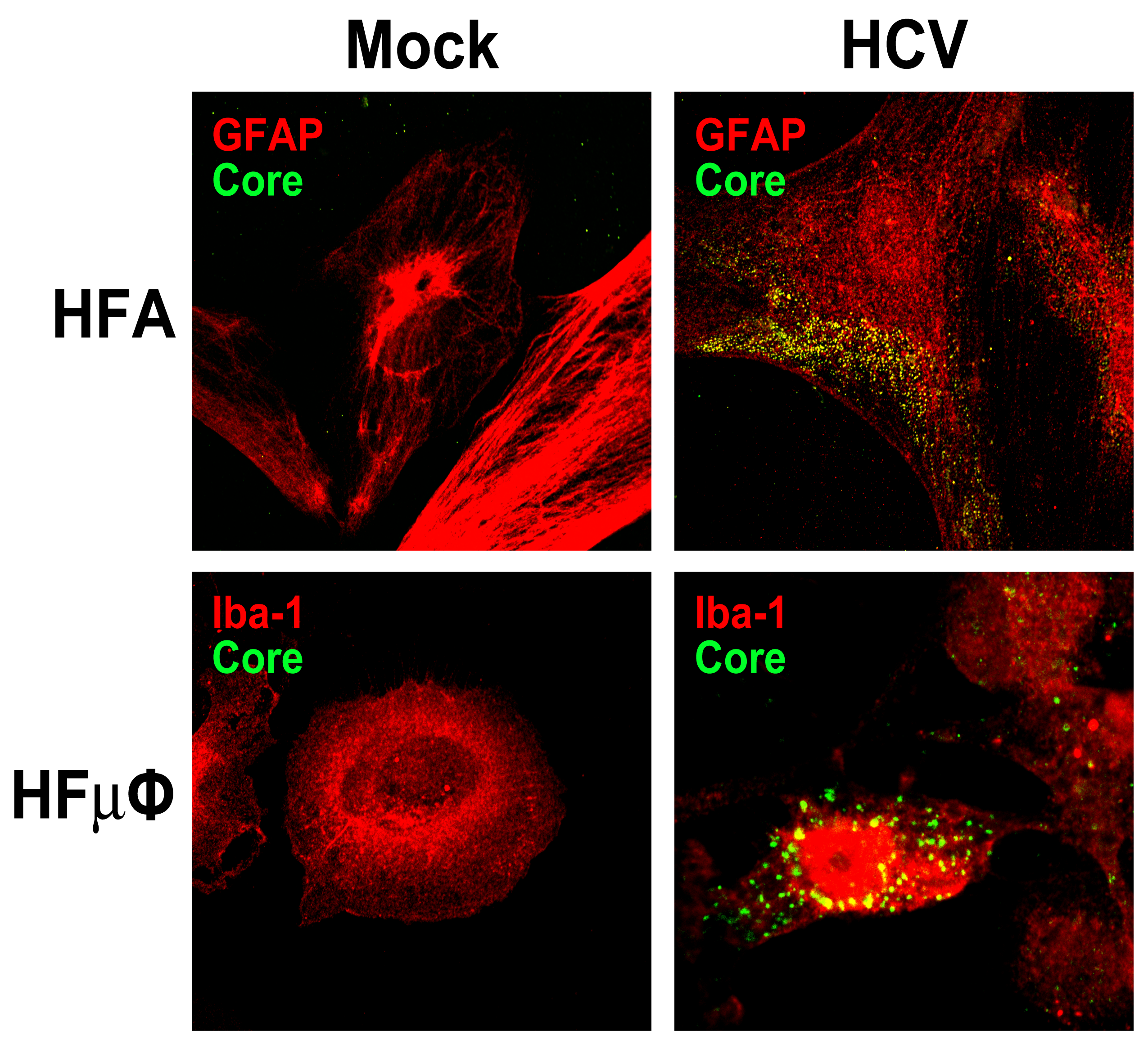

Supplement: Figure S1 — Primary human astrocytes and microglia were permissive to infection by HCVcc. At day 3 post-infection, HCV core protein immunoreactivity (green) was colocalized with GFAP or Iba-1 (red) in HCVcc-infected primary human fetal astrocytes (HFA) and microglia (HFµΦ) but not in mock-infected cells. (original magnification 630x) (4.39 MB TIF) [file pone.0012856.s002.tif]

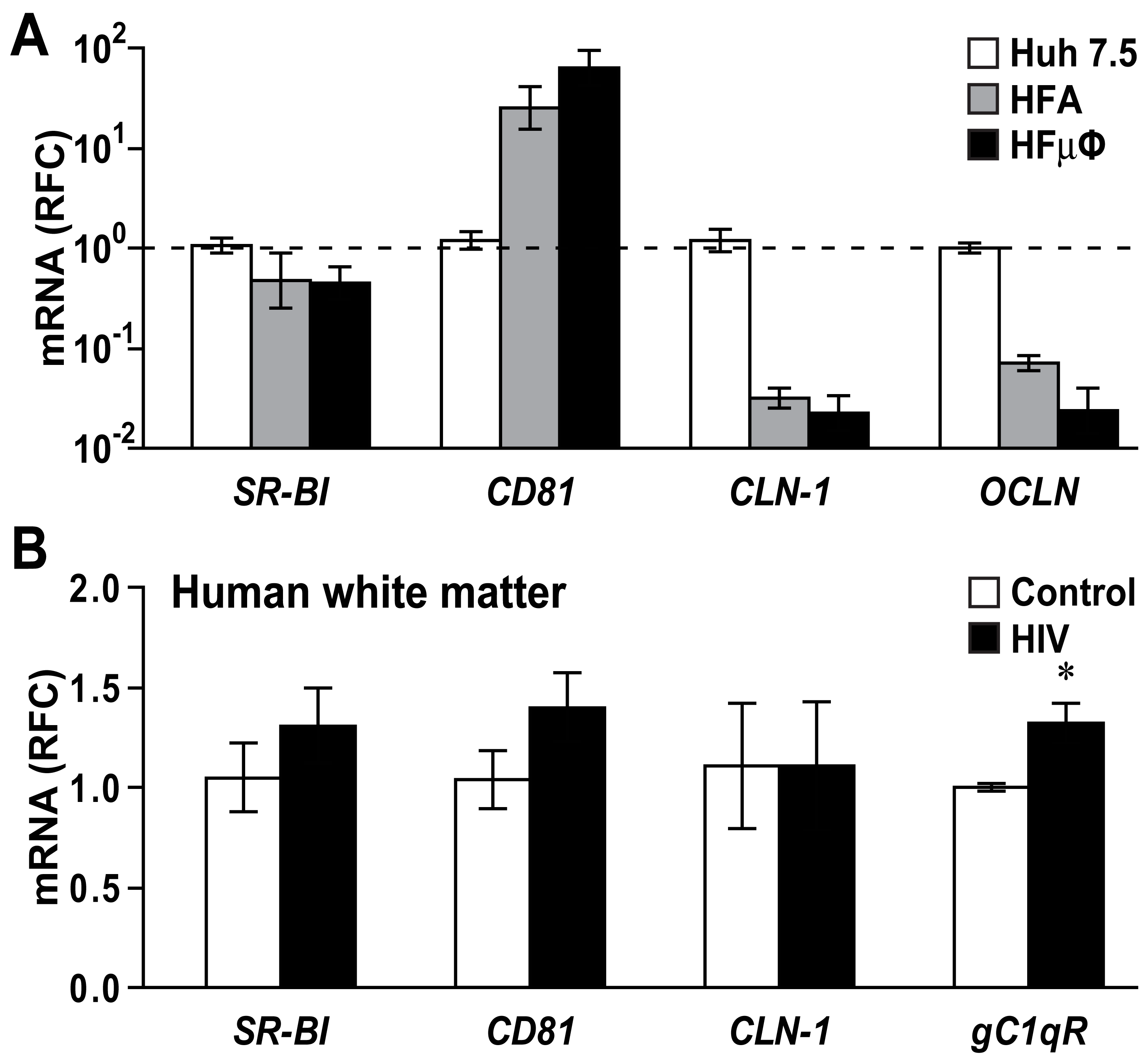

Supplement: Figure S2 — Expression of HCV entry receptors in glial cells and brains. (A) Transcript expression of HCV entry receptors were determined by real time RT-PCR. Primary human microglia and astrocytes expressed all known HCV entry receptors, albeit at the lower levels than Huh 7.5 hepatoma cell line. (B) Similar levels of HCV entry receptors were found in white matter tissues from control and individuals with HIV encephalitis (HIV) while the expression of the gC1qR transcript was elevated in HIV compared to control (n = 5, * p<0.05). Data represent mean ±SEM for three or more independent experiments. (0.81 MB TIF) [file pone.0012856.s003.tif]
